# Optimal resistance exercise training parameters for stroke recovery: A protocol for a systematic review

**DOI:** 10.1371/journal.pone.0295680

**Published:** 2023-12-07

**Authors:** Kenneth S. Noguchi, Kevin Moncion, Elise Wiley, Ashley Morgan, Eric Huynh, Marla K. Beauchamp, Stuart M. Phillips, Lehana Thabane, Ada Tang

**Affiliations:** 1 School of Rehabilitation Science, McMaster University, Hamilton, ON, Canada; 2 Department of Kinesiology, McMaster University, Hamilton, ON, Canada; 3 Department of Health Research Methods, Evidence & Impact, McMaster University, Hamilton, ON, Canada; 4 St Joseph’s Healthcare Hamilton, Hamilton, ON, Canada; 5 Faculty of Health Sciences, University of Johannesburg, Johannesburg, South Africa; The Second Affiliated Hospital of Shandong First Medical University, CHINA

## Abstract

**Background:**

Stroke impacts nearly 14 million people annually. Muscle strength and physical function are often affected by stroke and important determinants of stroke recovery. Resistance exercise training (RT) has been shown to improve muscle strength, but RT prescriptions may be suboptimal for other aspects of stroke recovery. Parameters such as frequency, intensity, type, and duration may influence the effectiveness of RT interventions but have not been systematically evaluated.

**Objectives:**

1) To determine the effects of RT on stroke recovery, and 2) to examine the influence of RT parameters on intervention effects.

**Eligibility criteria:**

Randomized controlled trials examining the effects of RT will be eligible for this systematic review if they: 1) included only adults with stroke or transient ischemic attack, 2) compared RT to no exercise or usual care, and 3) did not apply a co-intervention.

**Study selection:**

Eight databases (MEDLINE, EMBASE, EMCARE, AMED, PsychINFO, CINAHL, SPORTDiscus, and Web of Science) and 2 clinical trials registries (ClinicalTrials.gov and the WHO International Clinical Trials Registry Platform) will be searched from inception. Two independent pairs of authors will compare titles, abstracts, and full-text reports against the eligibility criteria. Conflicts will be resolved by consensus or third author.

**Main outcome measures:**

The construct of interest is stroke recovery. An advisory group of clinicians, researchers, and partners with lived experience of stroke will be consulted to determine specific outcome measures of interest, and to rank their relative importance. We expect to include measures of physical function, strength, cognition, and quality of life. Random-effects meta-analyses will be used to pool results for each outcome across studies, and RT parameters (frequency, intensity, type, and duration) will be used as covariates in meta-regression analyses.

**Conclusion:**

The results of this review will inform the optimal RT prescription parameters for promoting stroke recovery.

## Introduction

Stroke is a leading cause of disability worldwide and affects nearly 14 million people each year [1]. Many individuals with stroke experience losses in skeletal muscle mass [2], which manifest clinically as losses in muscle strength [3] and walking independence [4]. Compromised physical function after stroke initiates a cycle of physical inactivity and accumulation of risk factors [5], such that nearly a quarter of individuals will experience a second stroke within 5 years of their index event [6]. Therefore, interventions that break the cycle of inactivity, accumulation of risk, and subsequent recurrent events, are imperative for this population [7].

The American Heart Association recommends resistance training (RT) to retain skeletal muscle mass, improve strength and prevent declines in physical capacity in individuals with stroke [7]. Small scale randomized controlled trials have shown that RT may improve cardiac function, blood glucose regulation and regulate circulating cholesterol levels after stroke [8, 9]. Previous systematic reviews have found that RT can improve skeletal muscle strength but without unclear effects in other aspects of stroke recovery (e.g., functional mobility, walking ability, overall disability status) in people with stroke [10–12]. Despite being recommended in stroke clinical practice, researchers and clinicians are left with much debate on the efficacy of RT for promoting recovery after stroke [10–12].

Variability in RT parameters (i.e., frequency, intensity, type, and duration) across studies may explain the heterogeneity observed in training effects. Systematic reviews in the general population have found that higher frequencies and intensities of RT may elicit greater improvements in outcomes such as muscle strength and hypertrophy [13, 14]. However, no systematic reviews have examined whether the heterogeneity in effects can be explained by variability in RT parameters used after stroke. Clinical practice guidelines for stroke broadly recommend 1–3 sets of 10–15 repetitions at 50–80% 1-repetition max, 2–3 times per week, involving major muscle groups with a variety of equipment to improve everyday function [7]. The broad range of RT parameters reflects the absence of systematic review-level evidence to provide more specific recommendations, which may contribute to limited uptake to clinical practice guidelines in the community [15]. There is a critical need for a systematic review to determine the appropriate frequency, intensity, type, and duration of RT to optimize stroke recovery.

### Research questions

The research questions for this systematic review are twofold:

1. What are the effects of RT on stroke recovery?
2. Are prescribed RT parameters (frequency, intensity, type, and duration) associated with stroke recovery?

## Materials and methods

This systematic review protocol has been prospectively registered (PROSPERO: CRD42023414077) and is reported according to the Preferred Reporting Items for Systematic reviews and Meta-Analysis Protocols (PRISMA-P, S1 File) [16].

### Review team

The review team will consist of 9 academic members (5 trainees and 4 faculty members) and up to 7 community members. The academic members have research expertise in several areas including stroke rehabilitation, aging, musculoskeletal health, statistical analysis, clinical trial, and systematic review methodology. Four academic members are physical therapists with clinical backgrounds in stroke and aging.

We will also recruit 5–7 community members with lived experience of stroke who will form our community advisory group and will engage with the review in several capacities. Community advisory group members will be recruited by invitation from local stroke groups and will be diverse with respect to stroke experience (i.e., level of disability and type of stroke), biological sex, race, and age. All advisory group members will be involved continuously during the review according to the ACTIVE (Authors and Consumers Together Impacting on eVidencE) Framework, described by Pollock and colleagues [17]. The framework details 12 stages in the review process, where partners may look to contribute. Generally, they will be involved in: planning methods, selecting evidence, analysis, interpretation, writing, and knowledge translation activities. Details of the engagement are described below. Community advisory group members will also be invited as co-authors on the final manuscript.

### Eligibility criteria

We will include randomized trials examining the effects of RT compared to no exercise or usual care in people living with stroke, transient ischemic attack (TIA), or cerebrovascular accident. Studies will be included if they 1) included only adults (≥18 years old) with a primary diagnosis of stroke, TIA or cerebrovascular accident, 2) used any form of RT (functional, circuit, or traditional RT) as defined by the American College of Sports Medicine [18] (i.e., a form of exercise designed to improve muscular fitness by exercising a muscle or muscle group against external resistance), 3) compared RT to no exercise, usual care, or conventional therapy, and 4) reported at least 1 exercise prescription parameter (frequency, intensity, type, duration of intervention and/or sessions).

The exclusion criteria are as follows: i) not primary research articles (e.g., thesis/dissertations, systematic reviews, secondary data analyses), and ii) trials that combined RT with another form of therapy or co-intervention that is currently recommended [19] to improve physical function in people with stroke. Ineligible co-interventions include: Mental imagery, functional electrical stimulation, constraint-induced movement therapy, mirror therapy, sensory stimulation, virtual reality, bilateral arm training, non-invasive brain stimulation, treadmill-based gait training, robot-assisted training, Tai-Chi, other forms of exercise therapy. Nutritional and/or pharmacological co-interventions will also be ineligible. Aerobic and stretching exercises will be permitted if they are only used for warm-up and/or cool-down purposes. Trials that included balance and functional exercises will not be considered co-interventions, as they are commonly used in functional strength training interventions.

There will be no restrictions for time post-stroke. We will include studies from the acute (0 to 1 week post-stroke), subacute (1 week to 6 months post-stroke), and chronic phases of stroke recovery (>6 months post-stroke) [20]. However, given the heterogeneity in definitions of “usual care” or “conventional therapy” during the chronic phases of stroke recovery, we will only include studies comparing RT to “usual care” or “conventional therapy” if the study population was in the acute to subacute phase of stroke recovery (i.e., less than 6 months), or if usual care was otherwise explicitly defined as no intervention. There will be no restrictions on the types of activities included in usual care or conventional therapy.

### Information sources & search strategy

Eight electronic databases (MEDLINE, EMBASE, EMCARE, AMED, PsychINFO, CINAHL, SPORTDiscus, and Web of Science) will be searched from inception for articles published in English. The search strategy was developed in consultation with a research librarian from McMaster University with experience in exercise-based interventions. A sample of the search strategy for MEDLINE can be found in Table 1. Additionally, we will search 2 clinical trial registries (ClinicalTrials.gov, and WHO International Clinical Trials Registry Platform) and perform hand-searches through Google Scholar’s “cited by” function, and through reference lists of included studies and relevant systematic reviews. We will search for studies using terms related to “Stroke or Transient Ischemic Attack” and “Resistance Training”. The construct of interest for this systematic review is stroke recovery, which will be defined by our partners before data extraction. As such we did not include any key terms related to specific outcomes of interest.

**Table 1:** Search strategy for MEDLINE.

1	exp brain ischemia/ or carotid artery diseases/ or cerebral small vessel diseases/ or cerebrovascular trauma/ or exp stroke/
2	(((brain or cereb*) adj2 infarct*) or stroke or cerebrovasc* dis* or cerebrovasc* accident or transient isch* attack or TIA).ti,ab,kw,kf.
3	stroke volume.ti,ab,kw,kf.
4	1 or 2
5	4 not 3
6	plyometric exercise/ or resistance training/ or circuit-based exercise/ or muscle strength
7	(((resist* or strength* or plyometric* or ballistic* or weight* or isometric* or isotonic* or eccentric* or circuit) adj3 (train* or exercise*)) or resist* train* or strength* exercise* or strength* train* or weight lift* or weight train* or weightlift* or resist* exercise or plyometric* train* or ballistic* train* or plyometric exercise* or ballistic exercise* or ballistic* or weightbear* exercise* or weight bear* exercise*).ti,ab,kw,kf.
8	6 or 7
9	5 and 8

### Study records

Four independent pairs of authors (KSN and one other) will pilot the study selection process with 100 titles and abstracts (i.e., 25 per independent pair) and 20 full-text articles (i.e., 5 per independent pair) using a pre-populated Microsoft Excel spreadsheet (Microsoft Corporation, Redmond, WA, USA). Next, independent pairs of authors will screen titles and abstracts and full-text articles using online systematic review management software (Covidence, Veritas Health Innovation Ltd, Melbourne, Victoria, Australia). Disagreements will be managed by consensus discussion or consultation with an author not involved in the disagreement.

Data from included records will be extracted using online systematic review management software (Covidence, Veritas Health Innovation Ltd, Melbourne, Victoria, Australia). Two authors will pilot the data extraction process with 5 included studies, and fields will be refined after discussion. Thereafter, 2 independent pairs of authors (KSN, and EW, AM or EH) will extract data using the updated extraction template. Any missing data will be handled by contacting the corresponding author(s), then by imputation, whenever possible. Discrepancies between authors will be resolved by consensus or consultation with a third author if disagreement persists.

### Data items

#### Demographic information

We will consult with our community advisory group members to ensure we are extracting all relevant data. At minimum, publication information (e.g., study title, trial registration number, first and corresponding authors, publication year and country), participant demographic information (e.g., sample size, proportion of female participants, age, time post-stroke, stroke severity), and study setting (i.e., hospital or community, virtual, at home or in-person) will be extracted. Details regarding the RT programs will also be extracted, including frequency (sessions per week), intensity (e.g., % 1-RM, or rating of perceived exertion), type (e.g., functional, circuit and/or traditional), duration (number of weeks), progression methods and exercise training setting (i.e., group or individual training).

#### Outcome measures

The construct of interest for this systematic review is stroke recovery. The review team will extract all outcomes related to stroke recovery from each trial. To operationalize the relevant outcomes to stroke recovery, the community advisory group members will work with the review team to collaboratively determine a core set of outcomes to include in the review. The members will undergo training to explain scientific jargon and outcome measures extracted from the review. They will attend five 90-minute meetings over the course of the review, wherein the lead author (KSN) will guide them through the review processes. We expect to consider measures of upper and lower-extremity physical function, strength, cognition and quality of life.

### Risk of bias assessment

We will use the Cochrane Collaboration revised risk of bias assessment (RoB-2) tool [21] to assess risk of bias in each study across 5 domains of: the randomization process, deviations from intended intervention, missing outcome data, measurement of the outcome, selection of the reported result. Risk of bias will be presented with either a summary plot or “traffic light” plot, generated by an application from McGuinness & Higgins [22]. Studies with an overall high risk of bias will be removed in sensitivity analyses. Two independent authors will use the RoB-2 assessment. Disagreements will be resolved by consensus discussion or by third author in disagreement persists.

### Data synthesis

Results will be synthesized qualitatively in-text and in tabular format. The review team will work with the community advisory group members to identify research gaps emerging from the included studies, with interest in equity-related gaps such as recruited participant’s sex, race and age. The gaps will be described in-text and in tabular format. All quantitative data will be synthesized using Stata SE, version 17.0 (StataCorp LLC, College Station, TX, USA). The accepted significance level for all analyses is set to *α* = 0.05.

To answer our primary research question of examining the effect of RT on stroke recovery, we will use random-effects meta-analyses, comparing 1) RT to no exercise and 2) RT to usual care across each outcome measure. Variables will be treated as continuous and effect estimates will be presented as mean differences whenever possible. Whenever possible, we will group measures by test. Otherwise, standardized mean differences (SMD) will be presented using Glass’s delta (Δ) for pooled outcome measures. For instance, if there are insufficient studies represented for certain outcomes, we will combine measures by upper- and lower-body outcomes and by construct (e.g., composite versus single-construct measures of physical function, muscle strength, muscle power, etc.). Cluster randomized controlled trials will be treated by multiplying the standard errors of treatment effects by the square root of the design effect, as outlined by the *Cochrane Handbook for Systematic Reviews of Interventions* [23]. If standard deviations (SD) of change scores are not provided, they will be imputed using the following formulae, per the *Cochrane Handbook* [23]:

$$Corr=\frac{{SD}_{Baseline}^{2}+{SD}_{Final}^{2}-{SD}_{Change}^{2}}{2\times {SD}_{Baseline}\times {SD}_{Final}}$$ (1)

$${SD}_{Change}=\sqrt{{SD}_{Baseline}^{2}+{SD}_{Final}^{2}-(2\times Corr\times {SD}_{Baseline}\times {SD}_{Final})}$$ (2)

For our secondary research question of examining the influence of RT parameters on treatment effects, we will conduct separate, univariable meta-regression analyses across 4 covariates: frequency, intensity, type, and duration of the RT program. Frequency (sessions per week) and duration (number of weeks) will be treated as continuous, the intensity will be treated as categorical using contemporary definitions of low, moderate, high intensity, and high velocity (i.e., power focused) as defined by the American College of Sports Medicine [18], and type (separated by traditional, functional, or circuit-based RT) also as categorical. Definitions for each parameter category are in Table 2. The credibility of statistically significant meta-regression analyses will be evaluated using the Instrument to assess the Credibility of Effect Modification Analyses (ICEMAN) [24].

**Table 2:** Definitions for each parameter category for meta-regression analyses.

Parameter	Definition
Frequency	Continuous, sessions per week
Intensity*	**High intensity:** ≥70% 1-RM, RPE ≥7/10 or equivalent **Moderate intensity:** 50–69% 1-RM, RPE 5-6/10 or equivalent **Low intensity:** <50% 1-RM, RPE <5/10 or equivalent **Power-focused intensity:** Any external load, high velocity
Type	**Traditional RT:** Exercises with a goal to increase muscle strength, designed in blocks of sets and repetitions for a single muscle group per exercise. **Functional RT:** Movements that simulate everyday activities over multiple planes of movement to develop multiple muscle groups **Circuit-based RT:** Groupings of exercises performed sequentially with no or minimal rest between exercises, with or without rest between rounds.
Duration	Continuous, weeks

*Determined from the American College of Sports Medicine [18]

Heterogeneity will be assessed first by visual inspection of the forest plots and chi-square test (i.e., Q statistic), where a statistically significant test indicates the presence of heterogeneity between studies. As the *Cochrane Handbook for Systematic Reviews of Interventions* [23] outlines, we will consider low, moderate, substantial, or considerable heterogeneity if I^2^ <30%, 30–60%, 60–75%, and >75%, respectively. For our primary research question, we will explore heterogeneity using univariable meta-regression analyses with 3 covariates: time post-stroke (3 levels: acute, sub-acute, and chronic stroke), age (continuous variable), and sex (3 levels, tertiles of low, moderate, and high proportion of females). Missing covariate data will be handled first by contacting the corresponding authors, then excluding trials if data is not present. Publication bias will also be examined by visual inspection of the funnel plot and Egger test, and sensitivity analyses will be performed by removing studies found to have small-study effects, defined as having high influence on funnel plot asymmetry (point lays outside of 95% confidence intervals).

### Certainty of evidence

We will use the Grading of Recommendations, Assessment, Development and Evaluation (GRADE) approach to assess the certainty of evidence based on 8 criteria: Risk of bias, inconsistency of results, indirectness of evidence, imprecision, large magnitude of effect, confounding effects, presence of a dose-response relationship, and publication bias [25]. Disagreements will be resolved by consensus discussion or by a third author in disagreement persists. For each outcome and analysis, the certainty of evidence will be considered either high, moderate, low, or very low.

Three review team members (KSN, KM, AM), who each have experience with RT program design and delivery, will perform the initial GRADE assessment. Thereafter, the community advisory group members with lived experience will be invited to provide their importance ranking. Each member will rate the importance of all included outcomes on a scale from 1 (of least importance) to 9 (of most importance). The median score across the 8 assessments will be taken. Scores for each outcome will be used in the GRADE assessment to label outcomes: *of limited importance* (median score: 1 to 3), *important but not critical* (median score: 4 to 6), or *critical* (median score: 7 to 9) [26].

## Ethics

This systematic review does not require ethics approval to conduct since data is from available sources and we are not requesting individual participant data. However, some ethical considerations may arise when working with people with lived experience. We will conduct our partnership with community advisory group members ethically by ensuring their involvement is active, ongoing, and clearly defined. From the onset of this partnership, we commit to (1) collaboratively develop their specific roles and responsibilities, (2) foster an open and inclusive environment where concerns and feedback are always welcomed, and (3) always consider accessibility (educational, cultural, and disability-related) in our activities. Moreover, to ensure a diverse group of people with lived experience are included, we will meet with candidate partners individually to better understand each person’s perspectives and capacity to contribute. We will aim to recruit individuals with diverse backgrounds and varying levels of stroke-related disability to inform our research.

## Discussion and dissemination

Several systematic reviews have shown that RT is a powerful stimulus for improving muscle strength, yet with unclear effects on other aspects of stroke recovery like physical function [10–12]. Current recommendations for RT prescription remain broad and unspecific, presenting a potential barrier for clinicians implementaing RT into practice. There is an untapped opportunity to determine the appropriate RT parameters, such as frequency, intensity, type, and duration for RT in people with stroke, which can help guide stroke clinical practice and inform clinical practice guideline development in the future.

Ours will be the first systematic review to directly compare different RT parameters in people with stroke using a meta-regression approach. Moreover, this will be the first review in this area of research to meaningfully integrate the perspectives of people with lived experiences of stroke throughout the review process. We expect that the contributions of our review team community advisory group members will add substantially to the literature, as they will help identify outcomes for stroke recovery that are meaningful to persons with lived experience and identify gaps in research for future studies to address. Furthermore, we will ask our advisory group members for feedback on visual dissemination materials and we will ensure they have opportunities to share findings in the community. Our goal is to provide robust data to support clear recommendations for RT parameters in upcoming clinical practice guidelines for stroke rehabilitation and promote widespread dissemination and implementation among people with stroke, clinicians and rehabilitation scientists.

## Supporting information

S1 File PRISMA-P checklist. (DOCX)
